# Progressive Deep Learning Framework for Recognizing 3D Orientations and Object Class Based on Point Cloud Representation †

**DOI:** 10.3390/s21186108

**Published:** 2021-09-12

**Authors:** Sukhan Lee, Yongjun Yang

**Affiliations:** 1 Artificial Intelligence Department, Sungkyunkwan University, Suwon 16419, Korea; 2 School of Information & Communication Engineering, Sungkyunkwan University, Suwon 16419, Korea; yongjun823@skku.edu

**Keywords:** orientation representation, 3D point cloud, 3D object, progressive learning, association network

## Abstract

Deep learning approaches to estimating full 3D orientations of objects, in addition to object classes, are limited in their accuracies, due to the difficulty in learning the continuous nature of three-axis orientation variations by regression or classification with sufficient generalization. This paper presents a novel progressive deep learning framework, herein referred to as 3D POCO Net, that offers high accuracy in estimating orientations about three rotational axes yet with efficiency in network complexity. The proposed 3D POCO Net is configured, using four PointNet-based networks for independently representing the object class and three individual axes of rotations. The four independent networks are linked by in-between association subnetworks that are trained to progressively map the global features learned by individual networks one after another for fine-tuning the independent networks. In 3D POCO Net, high accuracy is achieved by combining a high precision classification based on a large number of orientation classes with a regression based on a weighted sum of classification outputs, while high efficiency is maintained by a progressive framework by which a large number of orientation classes are grouped into independent networks linked by association subnetworks. We implemented 3D POCO Net for full three-axis orientation variations and trained it with about 146 million orientation variations augmented from the ModelNet10 dataset. The testing results show that we can achieve an orientation regression error of about 2.5° with about 90% accuracy in object classification for general three-axis orientation estimation and object classification. Furthermore, we demonstrate that a pre-trained 3D POCO Net can serve as an orientation representation platform based on which orientations as well as object classes of partial point clouds from occluded objects are learned in the form of transfer learning.

## 1. Introduction

Data representation is crucial when dealing with 3D objects. As far as data representation for 3D objects is concerned, there are three approaches available currently: (1) multiple 2D images from different perspectives, (2) voxel or octree representation and (3) 3D point cloud or mesh representation. Among them, 3D point cloud representation presents the most efficient means of representing 3D objects, featured with order-independence in its data structure. In addition, 3D point cloud representation is further supported by the availability of low-cost yet highly robust real-time RGB-D cameras. More significantly, the recent advancement of deep point networks, such as PointNet [1], FoldingNet [2] and their variants [3], demonstrates that 3D point clouds can be effectively processed by deep point networks for classification, segmentation and reconstruction with high accuracy and generalization power. In general, deep point networks employ a point-wise multi-layer mapping approach with shared weights, while choosing the maximum point values from individual axes of the mapped space to define the global features as a means of exploiting order-independence. Moreover, deep point networks can be trained based on a number of publicly available 3D object datasets, including ModelNet [4], PASCAL3D+ [5], ShapeNet [6], LineMod [7] and OCID [8], where 3D point clouds are either from object CAD models or from actual measurements. The deep point networks trained with 3D point cloud representation of 3D objects can effectively serve as a platform for the classification of 3D objects. Notably, however, as far as 3D objects are concerned, it is not only their classes, but also their poses, i.e., their orientations, that are important attributes to be represented. However, unlike object classes, 3D object orientations represent continuous variations about three independent rotational axes that pose a challenge in precisely identifying orientations using deep learning–based classification or regression. 

To date, deep learning approaches for 3D object recognition and orientation estimation are focused on relaxing limitations through a trade-off between precision and complexity. To deal with the trade-off, hybrid approaches are introduced such that the strength of deep learning approaches for object detection and recognition and that of conventional vision technologies for high precision orientation estimation are combined [9]. In other words, hybrid approaches seek for precision in orientation estimation at the expense of computational cost associated with conventional vision technologies. Should end-to-end deep learning approaches to orientation estimation be considered, they have to limit the number of orientation classes or the precision in regression to a manageable level [10]. Recently, a number of end-to-end deep learning approaches for 6D object pose estimation based on RGB and RGB-D data have been proposed. They show that end-to-end deep learning approaches can possibly achieve a sufficient level of accuracy in pose estimation, while taking full advantage of the processing speed provided by deep networks. They solve the problem of the precision–complexity trade-off by combining feature-induced regression, using local and global RGB or RGB-D features with deep iterative 6D pose refinement, supported by a powerful semantic segmentation of objects from a scene.

Despite the recent progress, the precision–complexity trade-off remains a fundamental issue for deep learning approaches in 6D pose estimation. In fact, this trade-off represents a general problem common to the estimation of multi-variate continuous functions through either classification or regression. Furthermore, should we include additional variations, such as occlusions, to represent 3D objects, the trade-off becomes even worse, due to the increased complexity. In this paper, we propose to solve the precision–complexity issue based on a progressive framework for learning the object class and three axes of orientation variations. The proposed framework is configured with four independent networks representing object class and three axes of orientations that are connected by in-between feature association subnetworks. The proposed framework progressively learns the object class and three axes of orientation variations with training samples limited only to pertinent variations. Instead, in-between feature association subnetworks learn to cover full data representations for independent networks. With the proposed progressive framework, we intend to develop a deep learning network that serves as a representation platform for 3D objects based on point cloud representation of 3D objects. As a platform, the proposed network is expected to be easily extended to learn partial point cloud representation of 3D objects, due to occlusion.

### 1.1. Related Work

Currently, the approaches proposed for the representation of 3D objects include the following: (1) multiple 2D images from different perspectives [11,12,13,14], (2) voxel representation and its variants, such as a hybrid grid–octree data structure [4,11,15,16,17,18], (3) 3D point cloud representation [19,20] and (4) mesh representation [21]. For deep learning approaches, voxel representation allows direct extension of the methodologies that are well established for 2D convolutional neural networks (CNNs). However, direct application of CNNs to voxel representation of 3D objects may suffer from computational cost, due to the inclusion of a large number of voxels with no actual contribution, although multi-level octree representation can reduce such computational cost to a certain degree [21]. On the other hand, point cloud representation is more efficient but requires special order independent processing, such as the one done by PointNet and FoldingNet, where the global geometric features are extracted based on a point-wise mapping with shared weights and a max selection operation. Note also the recent emergence of approaches to reinforce 3D object representation with structural or topological information based on graph or mesh convolutional networks [20,21,22].

Traditionally, 6D pose estimation is done by matching the extracted features with those of the ground truth object models [23]. Such traditional vision approaches may offer high precision in pose estimation, provided that a sufficient number of features can be extracted and matched for pose estimation. However, they suffer from high computational cost as a result of extracting and matching hand-crafted photometric and geometric features, besides the lack of robustness and generalization in dealing with variations. The recent advancement in deep learning networks offers an opportunity to overcome the limitation of traditional approaches by presenting a powerful platform for the detection, recognition and segmentation of objects in a cluttered scene [24,25,26]. Deep learning–based object detection, recognition and segmentation platforms are able to provide not only robustness and generalization in performance, but also fast processing speeds. The availability of such deep learning platforms enables the development of hybrid approaches [27], where deep learning approaches for object detection, recognition and segmentation are combined with traditional approaches to pose estimation so as to achieve high accuracy and speed at a reduced cost. Recently, end-to-end deep learning approaches for 6D pose estimation have emerged, where not only object detection, recognition and segmentation, but also 6D pose estimation are conducted by deep learning networks so that both accuracy and speed are at their maximum. For instance, RGB-based approaches determine 6D object poses either by directly regressing a quaternion representation of object orientations [28], by iteratively matching the rendered object view against the captured object image [12], or by predicting 3D coordinates of each object pixel through an auto-encoder generator, using a GAN framework. This is followed by iterative computation of the PnP algorithm with RANSAC [12]. In addition, RGB-D–based end-to-end deep learning network approaches are proposed for 6D pose estimation, in which pixel-wise embedding of color and point cloud, as well as the global feature representing both embedding, are generated and concatenated to regress pixel-wise 6D pose with the refinement of residual pose errors [29]. Lastly, it is worthwhile to introduce approaches extending pose estimation from an instance level to a category level, for instance, based on a pose-aware image generator trained by VAE for iterative optimization of object pose and shape [30], and a canonical representation of object categories with deep networks for estimating the object pose and size [31].

Recently, a progressive deep learning framework was proposed as a means of exploring the ability to transfer knowledge learned from prior tasks to a new task via lateral connections [32]. Such a progressive framework can be effective for learning multiple tasks or a complex task configured with multiple subtasks by correlating their embedded structure of data or knowledge. For instance, progressive frameworks have been applied to learning a variety of games in complex reinforcement learning domains [33] as well as modeling the acoustic features of noisy speech based on the knowledge transfer between different noise conditions [34]. Alternatively, progressive frameworks are adopted to recognize images having different visual complexities based on a set of network units activated sequentially with progressively increasing complexities, or to transfer knowledge between three paralinguistic tasks: speaker, emotion, and gender recognition, by exploiting how knowledge captured in one emotion dataset can be transferred to another [35]. Progressive deep learning frameworks have been reported to offer efficiency in learning with faster convergence and improvement in performance over conventional pre-training and fine-tuning with transfer learning [36].

### 1.2. Problem Statement and Proposed Approach

The fundamental issue to address here is how precise and accurate a deep learning network could be for estimating or predicting general three-axis orientations of 3D objects. This represents a general problem associated with deep learning approaches regarding their capability for approximating continuous functions with high dimensional input–output relationships. Recently, deep learning approaches to general three-axis orientation or 6D pose estimation of 3D objects based on classification and regression have shown considerable progress in their precision and accuracy toward the level offered by conventional hand-crafted feature engineering approaches [12,29,37]. However, further improvement in precision and accuracy, possibly to the level required by object manipulation tasks in various industrial applications, remains a necessity. Such improvement makes deep learning approaches highly preferable to conventional approaches for a wide range of applications with the advantages in robustness, generalization and computational speed. To tackle the above issue, we pay attention to how different ways to structure deep learning networks for estimating general three-axis orientations affect the performance in precision and accuracy. To be more specific, precision in orientation estimation relies on the number of classes to output, whereas accuracy in classification depends on the degree of data variations that individual output classes should generalize as well as the number of training data available for individual output classes. For higher precision and accuracy, we prefer defining a larger number of output classes, a smaller degree of data variations to generalize by each output class, and a larger number of training data available. On the other hand, for higher efficiency, we prefer a smaller number of output classes for structural simplicity manageable by available training data and computational power. As such, the structure of a deep learning network for orientation classification should be optimized in terms of the number of output classes, data variations associated with individual output classes, training data available as well as structural simplicity under optimal trade-off among precision, accuracy and efficiency. In particular, we need to address the issue caused by the exponential growth in the number of classes, as the orientation resolution represented by individual classes is increased for high precision estimation. Note that the above observation on precision and accuracy in orientation estimation in conjunction with classification structure can equally be applied to regression. This is because, in principle, they rely on the same structure for building embedding before outputs are trained in terms of regression or classification [37]. In fact, in this paper, we present both classification and regression for orientation estimation, where regression outputs are obtained as a weighted sum of class outputs with the weights given by class probabilities. Note that it is also possible to build a fully connected network on top of classification outputs to train for continuous orientation estimation, instead of a weighted sum of classification outputs with the weights from the probability distribution of output classes. We conjecture that classification-based regression has an advantage in that classification outputs play a role as control points in fitting data into a continuous regression function, where the distances of the data to control points may be used for local refinement of the regression function. 

**Figure 1:**
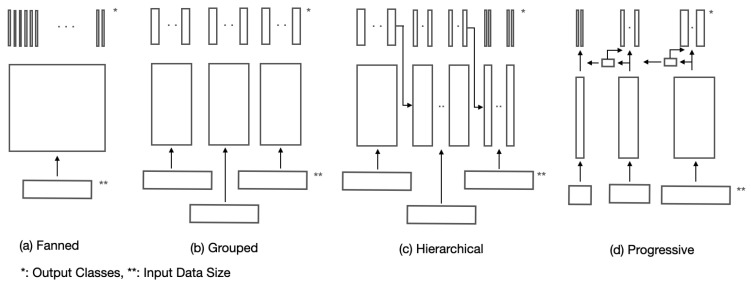
Feasible deep learning structures for estimating three-axis orientation estimation based on classification, including the proposed progressive structure.

The structure of conventional deep learning approaches to orientation classification can be categorized into “fanned”, “grouped” and “hierarchical” (Figure 1).

By “fanned”, we mean that all individual three-axis orientation classes are separately represented as individual output classes [10,19,37]. By “grouped”, we mean that all individual three-axis orientation classes are clustered into three separate groups: x-, y-, and z-axis orientation class groups, where the x-axis group consists of only x-axis orientation classes with all y- and z-axis orientations clustered into x-axis orientation classes, and so on. By “hierarchical”, we mean that objects are classified hierarchically, e.g., in a hierarchy of x-, y- and z-axis classifications [9]. Table 1 shows the number of output classes, the data variations associated with individual output classes, the training data available for individual output classes as well as the simplicity in network structures associated with the above three conventional structures. 

Generally speaking, for classification with a small number of orientation classes, a fanned architecture may be adopted. However, for classification with a large number of orientation classes, a grouped structure may be preferred for network simplicity at the expense of some accuracy. In between, we may consider a hierarchical structure as a compromise. 

In this paper, we propose a “progressive” structure for deep learning–based orientation classification, as an alternative to the above conventional structures, that can handle a large number of orientation classes by a small number of output classes, yet with a reduced degree of data variations. The proposed progressive structure achieves this by progressively learning the embedding of x-, y- and z-axis orientation classes one after another and, at the same time, by progressively extending the degree of data variations associated with individual axes and learning association between the embedding of two-axis orientation classes in sequence along the progression. For example, as illustrated in Figure 1, first, one axis class outputs are trained to learn their embedding with other axes data variations fixed to their reference orientations. Then, another axis class outputs are trained to learn their embedding with the remaining axis data variations fixed as their reference orientation and, at the same time, to learn the association between the current and the prior axis embedding. Finally, the last axis class outputs are trained to learn their embedding while learning the association between the current and the prior axis embedding. Table 1 shows the comparison of the proposed progressive structure with the conventional ones in terms of the number of output classes, the data variations associated with individual output classes, the training data available for individual output classes as well as the simplicity in network structures. It indicates that the proposed progressive structure allows the number of output classes to be the same as that of a grouped structure, yet allows the degree of data variations to be the same as that of a hierarchical structure in such a way that high precision and accuracy in the orientation estimation, closer to a fanned structure, can be achieved with a simple network structure.

## 2. D Point Cloud Based Object Class and Orientation Estimation Network: 3D POCO Net

**Figure 2:**
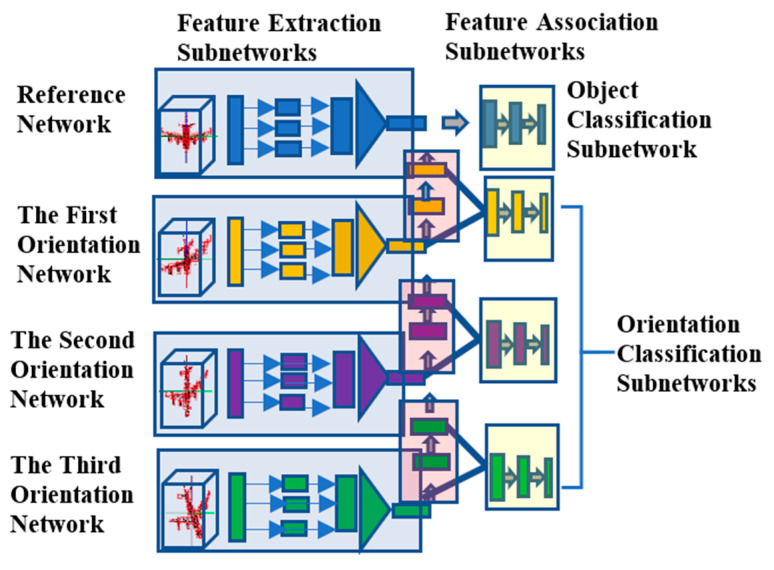
The proposed progressive framework with reference, first, second and third orientation networks.

As described in Section 1.2, we provide a deep learning network as a platform for representing object class and orientations in high precision and accuracy based on point cloud representation of 3D objects. To this end, a progressive framework for learning three axes of the orientation variations as well as the object class is designed and implemented based on independent networks that are connected by in-between feature association subnetworks (Figure 2). The proposed framework progressively learns the object class and three axes of orientation variations with training samples limited only to pertinent variations. Instead, in-between feature association subnetworks learn to cover full data representations for independent networks. The framework is based on the order-independent point cloud representation of PointNet for simplicity in implementation and computational efficiency. The progressive framework of networks thus implemented for object classification and orientation estimation is referred to here as the 3D Point Cloud Based Object Classification and Orientation Estimation Network or 3D POCO Net, in short form. 3D POCO Net is composed of the reference network, which outputs the classes associated with the 3D objects in their reference orientations and the three independent orientation networks, which generate their own orientations representing three consecutive rotations from the reference orientation. The reference network and the three orientation networks are linked by the association subnetworks that are trained to output the global features learned by the adjacent networks that they are linked to. Then, orientations of individual axes are estimated based on the weighted sum of orientation classes with the weight given as the class probabilities generated by the respective orientation networks. In the subsequent sections, we present the details of the network configuration and training procedure.

### 2.1. Network Configuration

Reference Network: The reference network is composed of the “feature extraction subnetwork” and “object classification subnetwork” (Figure 2). The aim of the feature extraction subnetwork is to extract the global features associated with the 3D objects in their reference orientations based on PointNet with no T-Net for orientation compensation. The feature extraction subnetwork is configured with five weight-sharing hidden layers in [64, 64, 64, 128, 1204] format for individual 3D point, where each layer is followed by non-linear activation and max-pooling operations. A 1024 dimension of the global feature vectors is then extracted by applying the element-wise max selection operation to the output of the last hidden layer. The resultant global feature vectors are then fed to the three-layered fully connected network of [512, 256, n_c_] configuration, with n_c_ representing the number of object classes. This object classification subnetwork adopts the Leaky ReLU activation and batch normalization [38] for all the layers, except for the last decision layer. In addition, a dropout layer with a dropout rate of 0.3 is used just before the decision layer. The Adam optimizer is used to optimize the network parameters.

Orientation Networks: Three orientation networks are configured in 3D POCO Net to generate their particular axes of rotations, such as roll, pitch and yaw angles. Each orientation network is composed of the “feature extraction subnetwork”, “orientation classification subnetwork” and “feature association subnetwork”. Apart from the reference network, each orientation network has the feature association subnetwork that is trained to generate the global features of the adjacent network. The feature association subnetwork is configured as a stack of fully connected layers of [1024, 768, 512, 768, 1024]. The orientation classification subnetwork concatenates the two outputs from its feature association and feature extraction subnetworks for use as input in order to obtain the probability of the predefined number of orientation classes as the output. The orientation classification subnetwork is configured as a stack of fully connected layers of [2048, 1024, 512, 256, n_o_], where no represents the number of predefined orientation classes. The orientation classification subnetwork adopts the Leaky ReLU activation and batch normalization for all the layers, except the last decision layer. Similarly, a dropout layer with a dropout rate of 0.3 is used just before the decision layer.

### 2.2. Training Procedure

**Figure 3:**
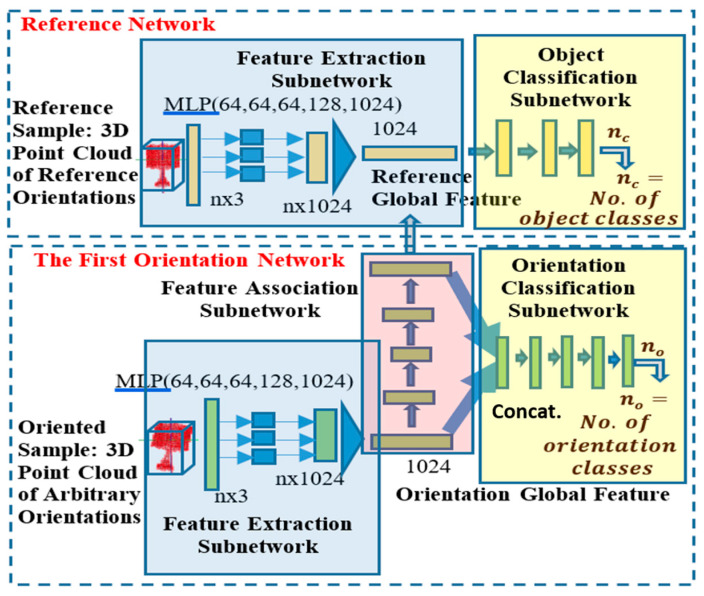
Illustration of the detailed configuration of 3D POCO Net with reference and first orientation networks.

The training of 3D POCO Net starts with training of the reference network for object classification based on the reference samples, i.e., the 3D object sub-dataset with the reference orientation as the input. Then, the trained reference network is kept fixed as the first orientation network is trained to learn its feature association and orientation classification subnetworks based on the first orientation samples, i.e., the 3D object sub-dataset generated by the first axis of rotation of the reference samples (Figure 3). In particular, this is followed by retraining the object classification subnetwork of the reference network in order to fine-tune based on the additional training samples available from the initial orientation samples. The same training procedure is repeated as the training proceeds to individual networks in the order of their association, except that retraining is applied to all the prior classification subnetworks one by one in the reverse order. The details of the training procedure are given in the following steps:

Step 1: The feature extraction and the object classification subnetworks of the reference network are trained using the reference sample data by optimizing the following loss function:

(1) $$L_{r}\left(\left\{y_{i}\right\},\left\{u_{i}\right\}\right)=\underset{i}{\sum }L_{cls}\left(f_{r}\left(r\left(y_{i}\right)\right),u_{i}\right)$$

where $y_{i}$ and $u_{i}$ represent the *i^th^* reference input sample and its object class, respectively; $r\left(y{\;}_{i}\right)$ represents the extracted global feature of $y_{i}$ from the feature extraction subnetwork; $f_{r}\left(r\left(y{\;}_{i}\right)\right)$ is the output of the object classification subnetwork; and $L_{cls}$ denotes SoftMax log classification loss.

Step 2: Once the training of the reference network is completed, the first orientation network is then trained, while the reference network trained is fixed, with the following objectives: 1) to make the output of its feature association subnetwork, $r^{\prime }\left(x_{i1}\right)$, $x_{i1}$ be the *i^th^* first orientation sample, which is equal to the output of the feature extraction subnetwork, $r\left(y{\;}_{i}\right),$ of the reference network; 2) to have its orientation classification subnetwork output, $f_{o}\left(r^{\prime }\left(x_{i1}\right),p\left(x_{i1}\right)\right),$ same as the true orientation class, $a_{i1}$, where the input of the orientation classification subnetwork is obtained by concatenating the output of its feature association subnetwork, $r^{\prime }\left(x_{i1}\right)$, and the output of its feature extraction subnetwork, $p\left(x_{i1}\right)$; and 3) to ensure that the first orientation sample, $x_{i1}$, also satisfies its object class constraint, i.e., $f_{r}\left(r^{\prime }\left(x_{i1}\right)\right)$ is equal to $u_{i}$. The feature association subnetwork, the orientation classification subnetwork and the feature extraction subnetwork of the orientation network are simultaneously trained by optimizing the following overall loss function:

(2) $$L_{o}\left(\left\{x_{i1}\right\},\;\left\{y_{i}\right\},\left\{u_{i}\right\},\left\{a_{i1}\right\}\right)=\underset{i}{\sum }L_{cls}\left(f_{o}\left(r^{\prime }\left(x_{i1}\right),p\left(x_{i1}\right)\right),a_{i1}\right)+\;\beta *\underset{i}{\sum }\sqrt{{\left|\left|r^{\prime }\left(x_{i1}\right)-r\left(y_{i}\right)\right|\right|}_{2}}+\left(1-\beta \right)*\underset{i}{\sum }L_{cls}\left(f_{r}\left(r^{\prime }\left(x_{i1}\right)\right),u_{i}\right)$$

where β represents the weight that balances the contributions of the feature association error and the object classification error. The loss is minimized based on the stochastic gradient decent optimization.

Step 3: The refinement of the object classification subnetwork of the reference network is then followed based on the first orientation sample data available from Step 2, while all the parameters of other subnetworks are kept fixed. Specifically, the dataset for refining the object classification subnetwork is created by randomly mixing all the pair-wise data, $\left\{r\left(y{\;}_{i}\right),\;u_{i}\right\}$ and {$r^{\prime }\left(x_{j1}\right)$, $u_{j}$}, representing the global features and their object classes of the reference samples and the first orientation samples, respectively, in order to form {$g_{k},\;u_{k}$}= $\left\{r\left(y_{i}\right),\;u_{i}\right\}\;U${$r^{\prime }\left(x_{j1}\right),u_{j}$ }, k = either i or j. The object classification subnetwork of the reference network is then retrained by optimizing the following loss function:

(3) $$L_{o}\left(\left\{g_{k},\;u_{k}\right\}\;\right)=\underset{k}{\sum }L_{cls}\left(f_{r}\left(g_{k}\right),u_{k}\right)$$

Step 4: Once the training of the first orientation network is completed, the second orientation network is trained in the same way as the first orientation network, and the second orientation sample dataset is generated by rotating the first orientation sample dataset about the second axis of rotation. First, with the first orientation network and the already trained reference network being kept fixed, the feature association subnetwork and the orientation classification subnetwork of the second orientation network are trained first by making their outputs, $r^{\prime }\left(x_{i2}\right)$ and $f_{o}\left(r^{\prime }\left(x_{i2}\right),p\left(x_{i2}\right)\right),$ become equal to the output, $p\left(x_{i1}\right)$, of the feature extraction subnetwork of the first orientation network and the true second orientation class, $a_{i2},$ respectively. However, the training of the second orientation network should ensure that not only the second orientation sample, $x_{i2}$, satisfies its first orientation class, i.e., $f_{o}\left(r^{\prime }\left(\;x_{i1}=r^{\prime }\left(x_{i2}\right)\right),\;r^{\prime }\left(x_{i2}\right)\right)$ = $a_{i1}$, but also that it satisfies its object class, i.e., $f_{r}\left(r^{\prime }\left(\;x_{i1}=r^{\prime }\;\left(x_{i2}\right)\right)\right)$ = $u_{i}.$ Therefore, the training of the second orientation network is based on the following loss function:

(4) $$L_{o}\left(\left\{x_{i2}\right\},\;\left\{x_{i1}\right\},\;\left\{a_{i2}\right\},\;\left\{a_{i1}\right\},\;\left\{y_{i}\right\},\left\{u_{i}\right\}\right)=\underset{i}{\sum }L_{cls}\left(f_{o}\left({r^{\prime }}^{\left(x_{i2}\right)},p\left(x_{i2}\right)\right),a_{i2}\right)+\;\alpha *\underset{i}{\sum }\sqrt{{\left|\left|r^{\prime }\left(x_{i2}\right)-p\left(x_{i1}\right)\right|\right|}_{2}}+\;\beta *\underset{i}{\sum }L_{cls}\left(f_{o}\left({r^{\prime }}^{\left(x_{i1}=\;r^{\prime }\left(x_{i2}\right)\right)},\;\;r^{\prime }\left(x_{i2}\right)\right),a_{i}\right)+\;\gamma *\underset{i}{\sum }L_{cls}\left(f_{r}\left({r^{\prime }}^{\left(x_{i1}=\;r^{\prime }\left(x_{i2}\right)\right)}\right),u_{i}\right)\;\mathrm{where}\;\alpha +\beta +\gamma$$

Step 5: The refinement of the first orientation classification subnetwork as well as the object classification subnetwork of the reference network is then followed by other subnetworks being kept constant. The refinement is based on all the sample data available from the second orientation sample data labelled with their first orientation classes and their object classes. For more details, refer to Step 3.

Step 6: The training of the third orientation network and the refinement of the first and second orientation classes and object classes are conducted in the same way as in Steps 4 and 5.

## 3. Experimental Verification

In this study, experiments were conducted to evaluate the performance of the proposed 3D POCO Net for 3D object classification and orientation estimation. For training and testing 3D POCO Net, the ModelNet10 dataset of 3D objects was used as the reference samples. First, 4905 3D object samples were obtained from the ModelNet10 dataset as reference samples, out of which 3994 and 911 samples were selected, respectively, for training and testing. Then, the reference samples from the ModelNet10 dataset were used to generate a large pool of data for three-axis orientation variations. Specifically, we generated a 3D object dataset for x-axis, y-axis and z-axis orientation variations by rotating the reference samples about x-axis, y-axis and z-axis by (α°, 0°, 0°), (α°, α°, 0°) and (α°, α°, α°), with α chosen as $3^{{}^{\circ}}$, $5^{{}^{\circ}}$ and $10^{{}^{\circ}}$ resolutions to cover 90° rotations. This led to a total of 152,055, 93,195 and 49,050 samples for ($3^{{}^{\circ}}$, 0°, 0°), ($5^{{}^{\circ}}$, 0°, 0°) and (10°, 0°, 0°), respectively; 4,713,705, 1,770,705 and 490,500 samples for ($3^{{}^{\circ}}$, 3°, 0°), ($5^{{}^{\circ}}$, 5°, 0°) and ($10^{{}^{\circ}}$, 10°, 0°), respectively; and 146,124,855, 33,643,395 and 4,905,000 samples for ($3^{{}^{\circ}}$, $3^{{}^{\circ}}$, 3°), ($5^{{}^{\circ}}$, $5^{{}^{\circ}}$, $5^{{}^{\circ}}$) and ($10^{{}^{\circ}}$, $10^{{}^{\circ}}$, $10^{{}^{\circ}}$), respectively. Total training time was about 16 h for training ($3^{{}^{\circ}}$, $3^{{}^{\circ}}$, 3°), ($5^{{}^{\circ}}$, $5^{{}^{\circ}}$, $5^{{}^{\circ}}$) and ($10^{{}^{\circ}}$, $10^{{}^{\circ}}$, $10^{{}^{\circ}}$) resolutions simultaneously up to 3200 epochs with 4 GPUs (RTX 2080 Ti). The average running time for ($3^{{}^{\circ}}$, $3^{{}^{\circ}}$, 3°), ($5^{{}^{\circ}}$, $5^{{}^{\circ}}$, $5^{{}^{\circ}}$) and ($10^{{}^{\circ}}$, $10^{{}^{\circ}}$, $10^{{}^{\circ}}$) resolution was about 0.01 sec. for each. 

Here, orientation classes are defined based on the resolution of orientation variations. However, the orientation estimation is done by the weighted sum of the orientation classes with the weights from the probability distribution of individual orientation classes so as to obtain continuous orientation estimation by regression. In addition, as a means of quantifying the performance of orientation estimation, we defined the following two performance indices, (1) the mean absolute error applied to the total testing samples, MAE-T, and (2) the mean absolute error applied only to the misclassified testing samples, MAE-F, as follows:

(5) $$\mathrm{MAE}\text{-}T=\frac{1}{N}\sum_{i}^{N}|orientation\_angle_{i}-u_{i}|$$

(6) $$\mathrm{MAE}\text{-}F=\frac{1}{M}\sum_{j}^{M}|orientation\_angle_{j}-u_{j}|$$

where $u_{i}$, $u_{j}$, *N* and *M* represent the ground truth orientation angles and the number of total and misclassified samples, respectively. 

For implementation, TensorFlow on TITAN X Pascal GPU is used. In training, the Adam optimizer is used with the mini-batch size of 32, at a learning rate of 0.001. 

### 3.1. Training and Testing of Reference Subnetwork for Object Classification Based on Samples with Reference Orientations

The reference network is trained and tested for object classification, using 3994 samples for training and 911 samples for testing. These samples serve as reference samples with reference orientations such that they are subjected to progressive rotation about z-axis, y-axis and x-axis in order to generate a larger pool of samples representing orientation variations based on the progressive framework. Note that it is free to choose the order of progressive rotations. We achieved 97.6% accuracy in object classification for the reference subnetwork with the reference samples (Table 2).

### 3.2. Training and Testing of the 1st Orientation Subnetwork for z-Axis orientation Variations

**Figure 4:**
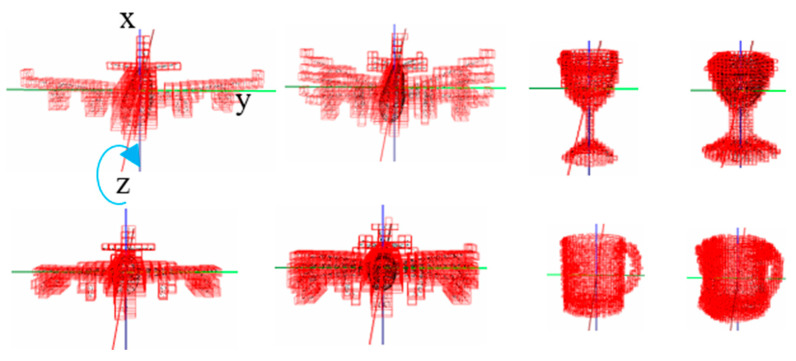
Examples of z-axis orientation variations (even column) by rotating reference orientation variations (odd column) around z-axis by $-10{}^{\circ},0^{{}^{\circ}},\;10{}^{\circ}$.

After training of the reference network is done, the first orientation network is trained and tested by defining 31, 19 and 10 orientation classes, respectively, with $3^{{}^{\circ}}$, $5^{{}^{\circ}}$ and $10^{{}^{\circ}}$ resolutions. To this end, we augmented the reference samples by rotating them about the z-axis by $3^{{}^{\circ}},\;5^{{}^{\circ}}$ and $10^{{}^{\circ}}$ resolutions, as illustrated in Figure 4 in the case of$\;10^{{}^{\circ}}$ resolution. 

This results in 123,814, 75,886 and 39,940 samples for training and 28,241, 17,309 and 9110 samples for testing, respectively, for $\;3^{{}^{\circ}},\;5^{{}^{\circ}}$ and$\;10^{{}^{\circ}}$ resolutions. Table 3 and Table 4 summarize the results. The result show that we can achieve less than $0.5^{{}^{\circ}}$ of MAE-T and 2.9° of MAE-F in regression with $3^{{}^{\circ}}$ precision in orientation classification, while achieving over 93% accuracy in object classification after retraining. 

### 3.3. Training and Testing of the 2nd Orientation Subnetwork for z-Axis and y-Axis Orientation Variations

**Figure 5:**
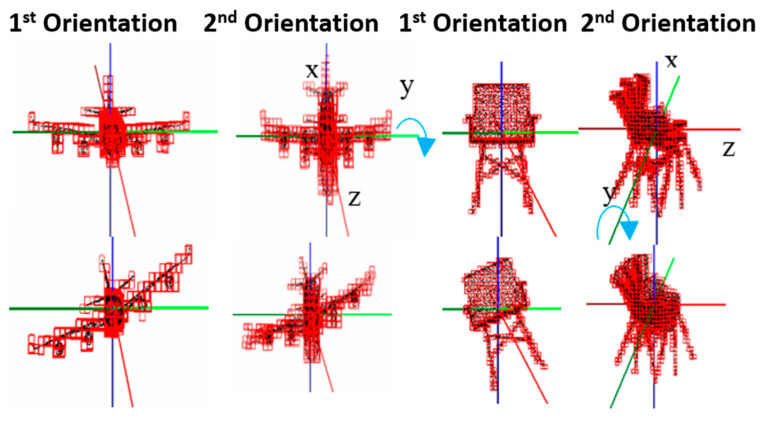
Examples of z-axis and y-axis orientation variations (even column) by rotating z-axis orientation variations (odd column) around y-axis by $-20^{{}^{\circ}}\;\mathrm{and}\;20{}^{\circ}$.

To train and test z-axis and y-axis orientation variations with the second orientation network, we rotate the 3D point cloud samples used for the first orientation network around the y-axis by $3^{{}^{\circ}}$, $5^{{}^{\circ}}$ and $10^{{}^{\circ}}$ resolutions and define 961, 361 and 100 orientation classes, respectively. Figure 5 illustrates the 1st and 2nd rotations. This results in 3,838,234, 1,441,834 and 399,400 samples for training and 875,471, 328,871 and 91,100 samples for testing, respectively, for $\;3^{{}^{\circ}},\;5^{{}^{\circ}}$ and$\;10^{{}^{\circ}}$ resolutions.

Table 5 and Table 6 show the accuracies of orientation classification and of regression with MAE-T and MAE-F for the second orientation network, as well as those of the retrained first orientation network. Table 7 shows the accuracy of object classification of the reference network after retraining. The results show that we can achieve less than $0.4^{{}^{\circ}}$ of MAE-T, 3.4 of MAE-F in orientation regression with $3^{{}^{\circ}}$ precision in orientation classification, while achieving about 93% accuracy for object classification after retraining. Although a slight degradation in performance is observed for the z-axis and y-axis orientation variations compared to only the z-axis orientation variation, the results summarized in Table 5, Table 6 and Table 7 suggest that the proposed progressive framework of learning orientation classes through data expansion and retraining works well.

### 3.4. Training and Testing of the 3rd Orientation Subnetwork for z-Axis, y-Axis and x-Axis Orientation Variations

**Figure 6:**
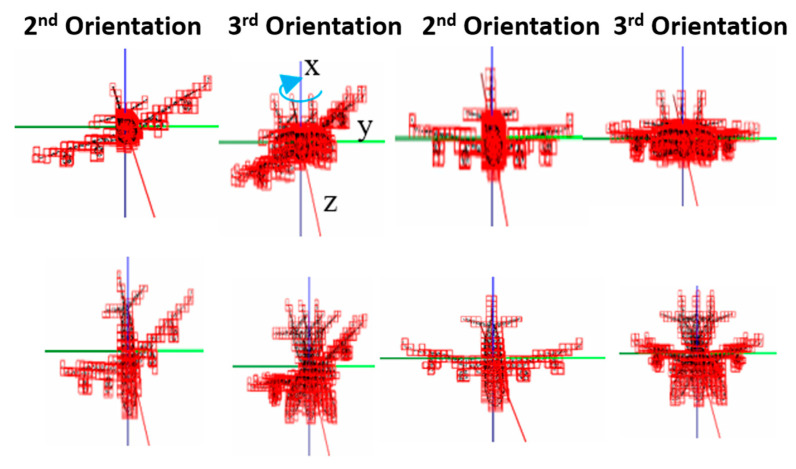
Examples of z-axis, y-axis and x-axis orientation variations (even column) by rotating z-axis and y-axis orientation variations (odd column) around x-axis by $-20^{{}^{\circ}},0^{{}^{\circ}},\;20{}^{\circ}$.

Using the z-axis and y-axis orientation variations as the reference samples, we rotate them again around x-axis with $3^{{}^{\circ}},\;5^{{}^{\circ}}$ and$\;10^{{}^{\circ}}$ resolutions to complete three-axis orientation variations for training and testing. Figure 6 illustrates 2nd and 3rd rotation examples.

**Figure 7:**
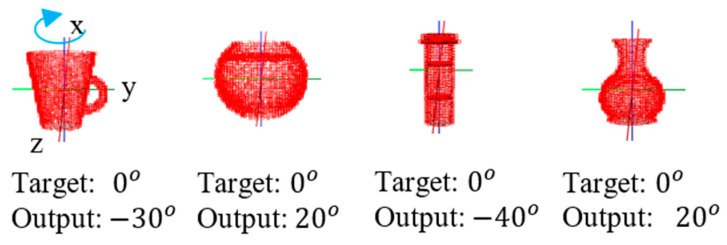
Examples of x-axis symmetric objects in ModelNet10 that cause incorrect orientation classification.

This defines 29,791, 6859 and 1000 classes, respectively, with $\;3^{{}^{\circ}},\;5^{{}^{\circ}}$ and$\;10^{{}^{\circ}}$ resolutions, while augmenting training and testing samples to 118,985,254, 27,394,846 and 3,994,000 samples for training and 27,139,601, 6,248,549 and 911,000 samples for testing, respectively, for $\;3^{{}^{\circ}},\;5^{{}^{\circ}}$ and$\;10^{{}^{\circ}}$ resolutions. Table 8, Table 9, Table 10 and Table 11 summarize testing accuracies in estimating general three-axis orientations and object class based on classification and regression for $\;3^{{}^{\circ}},\;5^{{}^{\circ}}$ and$\;10^{{}^{\circ}}$ resolutions. Table 8, Table 9 and Table 10 show that we can achieve the accuracies in x-, y- and z-axis orientation estimation, respectively, with 4.1°, 3.3° and 2.5° of MAE-T regression errors based on 3° resolution in orientation classification. Notice that the accuracies in x-, y- and z-axis orientation estimation based on 5° and 10° resolutions in orientation classification are not much different from those based on 3° resolution in orientation classification. On the other hand, Table 11 shows that we can achieve about 90% in object classification accuracy, similarly for $\;3^{{}^{\circ}},\;5^{{}^{\circ}}$ and$\;10^{{}^{\circ}}$ resolutions in orientation classification. Note that the performance of the three orientation networks after inclusion of the 3rd orientation network for x-axis rotation is somewhat reduced, compared to that of the two orientation networks before inclusion of the 3rd orientation network. This is partly due to the fact that the ModelNet10 dataset includes some objects that are rotation-symmetric about x-axis, as illustrated in Figure 7, such that the 3rd orientation network is unable to uniquely represent x-axis orientations for those objects. 

### 3.5. 3D POCO Net as a Representation Platform Applied to a Partial View 3D Point Cloud Data

**Figure 8:**
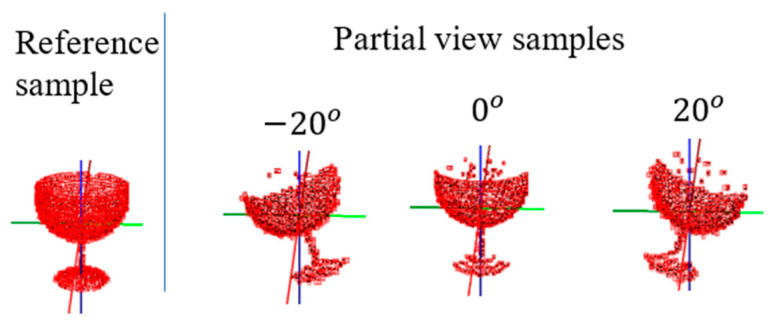
Partial view 3D point clouds created from full point cloud.

**Figure 9:**
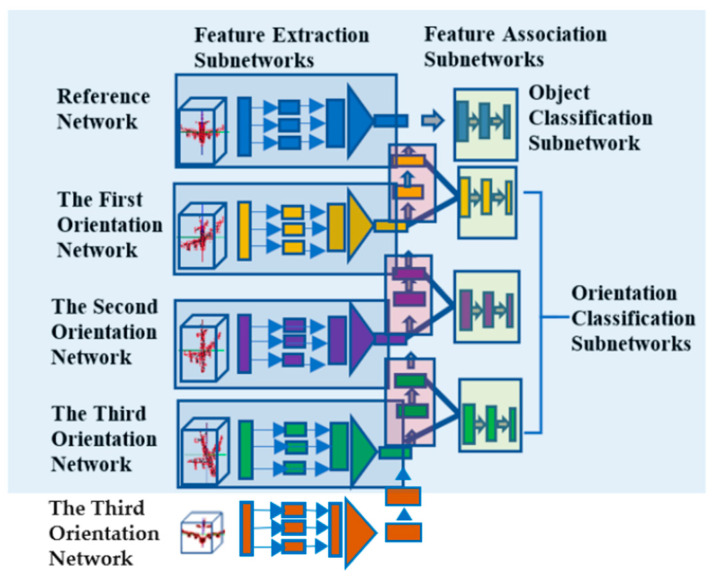
Expansion of 3D POCO Net to partial view data.

The pre-trained 3D POCO Net can be used as an orientation representation platform to which additional networks are attached to solve novel 3D pose estimation problems. To show this, we generated a partial view 3D point cloud dataset from the ModelNet10 dataset for use in partial point cloud–based object classification and orientation estimation (Figure 8). For classification of object class and orientations based on partial view 3D point clouds, we attached a PointNet, “Partial View Orientation Network,” to the third orientation network of 3D POCO Net through an association subnetwork, as shown in Figure 9 (in red marks).

The partial view orientation network and the association subnetwork attached to it are trained with the pre-trained 3D POCO Net, so that the two global features, i.e., the partial view orientation network and the third orientation network of 3D POCO Net match. Notably, in training, the loss function includes the errors from all the independent networks of 3D POCO Net.

Table 12 presents the results of testing partial view data with 10 orientation classes using $10^{{}^{\circ}}$ resolution around the z-axis. As shown, we achieved 1.73°, 0.25° and 0.3° of MAE-T in regression error for the respective z-, y- and x-axis orientation estimations, while achieving about 82% accuracy for object classification. This application demonstrates the modular extensibility of the proposed 3D POCO Net as a representation platform.

### 3.6. Discussion

The proposed progressive framework is trained to learn z-axis, y-axis and x-axis orientation variations progressively based on in-between feature associations with training samples limited only to pertinent orientation variations. Refer to Section 1.2 for the implication of the proposed progressive framework of learning object orientations in comparison with conventional deep learning approaches with fanned, grouped and hierarchical structures. We compared the performance of the proposed 3D POCO Net with that of the state-of-the-art approaches to orientation estimation (Table 13).

Due to differences in the training and testing datasets, as well as in the performance metrics, used by different approaches, direct comparison of performance is not feasible. However, Table 13 is intended to provide a general idea of where, among the current state-of-the-art approaches, the proposed 3D POCO Net with a progressive structure is positioned in terms of its methodology and performance. To further facilitate the performance assessment for the proposed approach, we extended the experiment with the ModelNet40 dataset to assess 3D POCO Net in terms of its effectiveness in handling a larger dataset. Out of total 12,308 samples in 40 object categories, we assigned 9840 and 2468 samples, respectively, to training and testing. To reduce computational burden, we defined output classes only with 10° orientation resolution for experiment. This leads to 10, 100 and 1000 output classes, respectively, defined for (10°, 0°, 0°): z-axis orientation variation, (10°, 10°, 0°): z- and y-axis orientation variations and (10°, 10°, 10°): z-, y- and x-axis orientation variations. Then, the ModelNet40 reference samples are augmented to 98,400 training and 24,680 testing samples for (10°, 0°, 0°), 984,000 training and 246,800 testing samples for (10°, 10°, 0°) and 9,840,000 training and 2,468,000 testing samples for (10°, 10°, 10°). The total training time is about 11 h for 2300 epochs, and the average running time for testing is 0.02 sec. The testing results are summarized in Table 14.

Table 14 shows that 3D POCO Net performs equally well with a larger dataset. The proposed framework offers a new approach for representing and estimating orientation variations, while enhancing the accuracy in orientation estimation with proper learning of in-between feature associations but with better efficiency.

## 4. Conclusions

In this paper, we propose a progressive deep learning framework for representing 3D objects in terms of their classes and orientations. The aim of the proposed framework is to offer high accuracy in regression and classification for three-axis orientations, yet with efficiency in the network structure. The unique features associated with the proposed framework include the following: (1) the proposed in-between association subnetworks learn to link between the networks that represent independent axes of variables so as to progressively reduce the constraint one after another; (2) independent networks are subject to retraining for refinement as the amount of data are increased with the progress of constraint relaxation. The experimental results based on the ModelNet10 dataset indicate that the proposed 3D POCO Net is effective for representing and estimating three axes of orientations with high accuracy yet with structural efficiency. For instance, the proposed 3D POCO Net is able to achieve a regression error of less than 3° in MAE-T, while achieving about 90% accuracy in object classification, for general three-axis orientation estimation and object classification with only 72 orientation output classes. The effectiveness of 3D POCO Net is further verified by applying it to general three-axis orientation estimation for a larger dataset, the ModelNet40 dataset, and for partial view-point cloud data. In particular, the latter indicates that a pre-trained 3D POCO Net can serve as an orientation representation platform to which partial point clouds from occluded 3D objects are linked for object classification and orientation estimation in a form of transfer learning. In future, further investigations will be conducted to enhance the performance by extending the applications to dealing with rotation-symmetric objects and occluded objects with a larger scale of various 3D object datasets.

## Figures and Tables

**Table 1:** Performance comparison of the proposed progressive structure with other typical structures for classification in terms of the number of output classes, degree of data variations and the number of training data per output class.

Structure	No. of Output Class	Degree of Data Variation per Output Class	No. of Training Data per Output Class
Fanned	N == M^3^	1	D/N(==M^3^)
Grouped	3M	3	D/M
Hierarchical Progressive	N + M^2^ + M 3M	3, 2, 1 for 1st, 2nd, 3rd Layer 1, 2, 3 for 1st, 2nd, 3rd Process	D/M, D/M^2^, D/M3 for 1st, 2nd, 3rd Layer D/M3, D/M2, D/M for 1st, 2nd, 3rd Process

N: Total no. of classes(N == M3), M: no. of classes for each axis, D: Total no. of training data.

**Table 2:** Object classification accuracy for the reference orientation with ModelNet10 data as reference samples.

	Reference Orientation
3D POCO Net	97.6%

**Table 3:** Orientation classification and regression accuracy for z-axis orientation variations from ModelNet10.

	TEST3 (3°)	TEST3 (5°)	TEST3 (10°)
Orientation Classification Rate	91.6%	93.9%	98.3%
Orientation Regression MAE-T	0.44°	0.6°	0.44°
Orientation Regression MAE-F	2.89°	5.0°	12°

**Table 4:** Object classification accuracy for z-axis orientation variations from ModelNet10.

	TEST3 (3°)	TEST3 (5°)	TEST3 (10°)
3D POCO Net	93.7%	93.8%	93.9%

**Table 5:** Orientation classification and regression accuracy for y-axis with z- and y-axis orientation variations for the second orientation network.

	TEST3 (3°)	TEST3 (5°)	TEST3 (10°)
Orientation Classification Rate	95.0%	96.8%	98.4%
Orientation Regression MAE-T	0.22°	0.23°	0.27°
Orientation Regression MAE-F	3.17°	5.4°	13.3°

**Table 6:** Orientation classification and regression accuracy for z-axis with z- and y-axis orientation variations for the first orientation network after retraining.

	TEST3 (3°)	TEST3 (5°)	TEST3 (10°)
Orientation Classification Rate	90.8%	93.8%	96.5%
Orientation Regression MAE-T	0.40°	0.47°	0.6°
Orientation Regression MAE-F	3.4°	5.9°	13.7°

**Table 7:** Object classification accuracy with z- and y-axis orientation variations from ModelNet10.

	TEST3 (3°)	TEST3 (5°)	TEST3 (10°)
3D POCO Net	93.0%	92.3%	92.1%

**Table 8:** Orientation classification and regression accuracy for x-axis with z-, y- and x-axis orientation variations for the third orientation network.

	TEST3 (3°)	TEST3 (5°)	TEST3 (10°)
Orientation Classification Rate	79.2%	85.2%	88.6%
Orientation Regression MAE-T	4.1°	4.2°	4.5°
Orientation Regression MAE-F	19.3°	23.8°	28.1°

**Table 9:** Orientation classification and regression accuracy for y-axis with z-, y- and x-axes orientation variations for the second orientation network after retraining.

	TEST3 (3°)	TEST3 (5°)	TEST3 (10°)
Orientation Classification Rate	79.2%	80.9%	82.3%
Orientation Regression MAE-T	3.3°	3.4°	3.6°
Orientation Regression MAE-F	6.9°	8.0°	16.2°

**Table 10:** Orientation classification and regression accuracy for z-axis with z-, y- and x-axis orientation variations for the first orientation network after retraining.

	TEST3 (3°)	TEST3 (5°)	TEST3 (10°)
Orientation Classification Rate	75.8%	79.1%	81.3%
Orientation Regression MAE-T	2.5°	2.8°	3.1°
Orientation Regression MAE-F	3.2°	5.8°	13.5°

**Table 11:** Object classification accuracy with z-, y- and x-axis orientation variations from ModelNet10.

	TEST3 (3°)	TEST3 (5°)	TEST3 (10°)
3D POCO Net	89.9%	90.2%	90.8%

**Table 12:** Orientation classification and regression accuracy of z-, y- and x-axis orientation variations in all three-axis classification from ModelNet10 with partial view samples in 10° resolution.

	First Orientation Network (z-Axis)	Second Orientation Network (y-Axis)	Third Orientation Network (x-Axis)
Orientation Classification Rate	92.5%	99.3%	99.3%
Orientation Regression MAE-T	1.73°	0.25°	0.3°
Orientation Regression MAE-F	13.2°	7.3°	23.9°

**Table 13:** Comparison of the proposed 3D POCO Net with the state-of-the-art approaches to object orientation estimation.

Reference	Approach	Dataset	No. of Classes/ Orientation Resolution	Classification Accuracy	Regression Error
Self-Supervised Learning of Point Clouds via Orientation Estimation [19]	Classification(Point Cloud-based /Fanned)	ModelNet-40	18 Spatial Vectors 32 Spatial Vectors	89.0% 90.3%	NA
Orientation-boosted Voxel Nets for 3D Object Recognition [10]	Classification (Voxel-based /Fanned)	ModelNet-10	18/20° (z-axis rot.)	89%	NA
3D Pose Regression using CNN [9]	Regression (Image-based/ Hierarchical)	Pascal 3D+	NA	NA	15.38°
Deep Learning for Spacecraft Pose Estimation [37]	Classification Based Regression (Image-based /Fanned)	Unreal Rendered Spacecraft On- Orbit (URSO)	32 × 32 × 32/30° (Euler Angles)	NA	7.4°
Our Approach: 3D POCO Net	Classification with Regression (Point Cloud-based/ Progressive)	ModelNet-10	31 × 31 × 31/3° 19 × 19 × 19/5° 11 × 11 × 11/10°	79.2% 85.2% 88.6%	2.5° 2.8° 3.1°

**Table 14:** Orientation regression and object classification accuracy of 3D POCO Net with the progressive variations of z-, y- and x-axis orientations and retraining, where 10° orientation resolution is applied to the ModelNet40 dataset.

10° Orientation resolution	Reference Net	z-axis Orient. Net	z- and y-axes Orient. Net		z-, y- and x-axes Orient. Net		
		z-axis	z-axis y-axis		z-axis y-axis x-axis		
Oient. Class. Rate	n/a	85.7%	83.2%	85.8%	75.9%	78.0%	82.7%
Ori. Regress. MAE-T	n/a	0.89°	1.2°	0.81°	5.4°	5.9°	7.2°
Ori. Regress. MAE-F	n/a	16°	15.1°	14.9°	16.2°	21.8°	30.9°
Object Class. Rate	87.3%	81.6%	80.2%		78.3%		

## Data Availability

Not applicable.
